# 3DSEM: A 3D microscopy dataset

**DOI:** 10.1016/j.dib.2015.11.018

**Published:** 2015-12-02

**Authors:** Ahmad P. Tafti, Andrew B. Kirkpatrick, Jessica D. Holz, Heather A. Owen, Zeyun Yu

**Affiliations:** a Department of Computer Science, University of Wisconsin-Milwaukee, WI, USA; b Department of Biological Sciences, University of Wisconsin-Milwaukee, WI, USA

**Keywords:** 3D microscopy dataset, 3D microscopy vision, 3D SEM surface reconstruction, Scanning Electron Microscope (SEM)

## Abstract

The Scanning Electron Microscope (SEM) as a 2D imaging instrument has been widely used in many scientific disciplines including biological, mechanical, and materials sciences to determine the surface attributes of microscopic objects. However the SEM micrographs still remain 2D images. To effectively measure and visualize the surface properties, we need to truly restore the 3D shape model from 2D SEM images. Having 3D surfaces would provide anatomic shape of micro-samples which allows for quantitative measurements and informative visualization of the specimens being investigated. The 3DSEM is a dataset for 3D microscopy vision which is freely available at [1] for any academic, educational, and research purposes. The dataset includes both 2D images and 3D reconstructed surfaces of several real microscopic samples.

**Specifications table**

**Table:** 

Subject area	*3D microscopy vision, biology, materials science, mechanical engineering.*
More specific subject area	*3D surface structure, 3D structural analysis.*
Type of data	*2D SEM images (.JPEG,.TIFF), 3D surface models (.OFF,.PLY).*
How data was acquired	*2D SEM images are captured by a Hitachi S-4800 field emission Scanning Electron Microscope (SEM). The 3D Shape models are created using the 3D reconstruction algorithm illustrated in*[2].
Data format	*Digital images, 3D shape models.*
Experimental factors	*Experimental setup along with its parameters described in*[2].
Experimental features	*Several qualitative and quantitative experiments showed in*[2]*. The results were promising.*
Data source location	*Milwaukee, Wisconsin, USA.*
Data accessibility	*The dataset is freely available at*[1]*for any academic, educational, and research purposes. More 2D SEM images and 3D surface models will be added into the dataset continuously.*

**Value of the data**

• Discovering 3D surface structure from SEM images would provide anatomic surfaces and allows informative visualization of the objects being investigated.
• To provide the current dataset, an optimized multi-view 3D SEM surface reconstruction algorithm is designed [2].
• Several experimental validations are performed on real microscopic samples as well as synthetic data. The quantitative and qualitative results are promising [2].
• Many research and educational questions truly require knowledge and information about 3D microscopic structures. The present dataset along with the algorithm would be helpful in this way.
• The current dataset which includes 2D SEM images and 3D surface models, and the underlying methodology may serve as a guide for 3D SEM surface reconstruction.
• The present work is expected to highlight the important roles and applications of 3D microscopy vision, particularly 3D surface reconstruction from SEM images, and open the doors for several interesting directions to advance the level of the research area.

## 1 Data

**Fig. 1:**
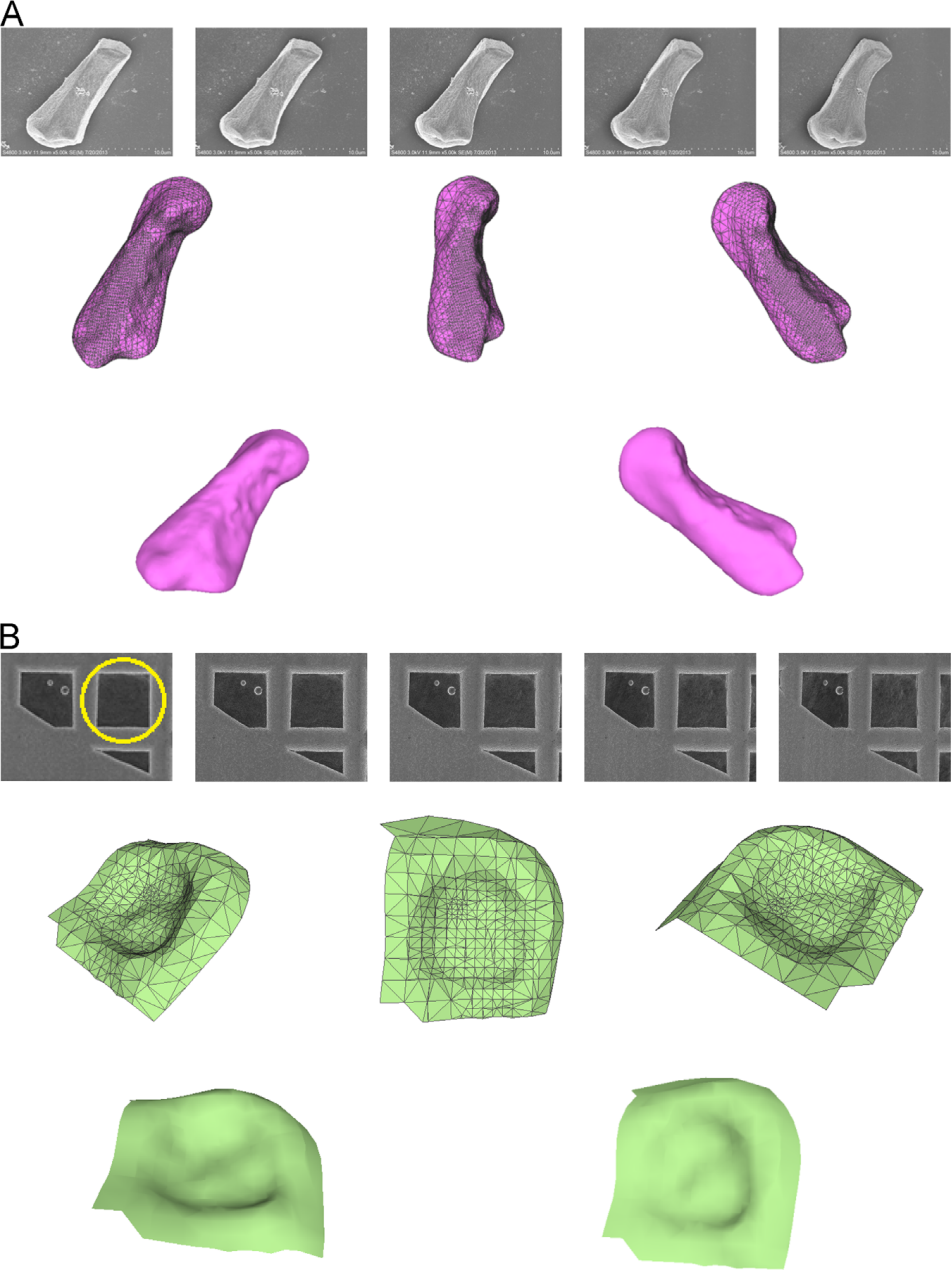
Qualitative visualization of a Tapetal cell of *Arabidopsis thaliana* (A), and TEM *copper grid* (B). The first row in each sample shows only a set of 2D images. The 3D surface reconstructed meshes and shape models are presented in the next rows respectively.

Dataset names and attributes are briefly presented in Table 1. Fig. 1 shows two samples of the entire dataset including 2D SEM micrographs and 3D reconstructed surfaces.

A Hitachi S-4800 Field Emission Scanning Electron Microscope is used to capture the micrographs. This microscope is equipped with a computer controlled 5 axis motorized stage capable of 360° of rotation with a tilt range of −5–70°. Sample manipulation, such as *Z*-position, tilt, and rotation of the stage, as well as image processing and capture functions are operated through the Hitachi PC-SEM software. The working distance that would give the required depth of focus is specified at the maximum tilt for every specimen at the magnification chosen for image capture. As the sample is tilted in successive 1° increments through the software, the image is centered manually by moving the stage in the *x*- and *y*-axes with the stage positioning trackball. The working distance and magnification are kept consistent in every captured image of the tilt series by changing the *Z*-axis position as required. Brightness and contrast are manually adjusted for consistency between micrographs, using the same structure in every image. The micrographs are acquired with an accelerating voltage of 3 kV, employing the signals from both the upper and lower secondary electron (SE) detectors. Readers interested in SEM imaging are referred to [4], [5] for further information.

The 3D surface models and their construction strategy are fully detailed in the paper [2]. At present, the 3DSEM dataset includes three different samples illustrated in Table 1. This dataset is an ongoing project in which further samples will be added to the dataset by near future. As we mentioned earlier, the 3DSEM dataset is freely available at [1] for any educational, research, and academic purposes.

## 2 Experimental design, materials and methods

3D surface reconstruction from a set of 2D images employs several computational technologies, including multi-view geometry, computer vision, machine learning, and optimization strategies to tackle the inverse problem going form 2D images to 3D surface models [6], [7]. The complete pipeline of our proposed optimized multi-view framework for 3D SEM surface reconstruction has six stages. At the first stage, a set of 2D SEM micrographs are taken by tilting the specimen across variant angles. The step requires SEM imaging styles, such as changing magnifications, tilting the specimen, employing SE or/and BSE detectors. We then detect the feature points in each 2D image in the set and estimate image motion based on a set of corresponding points. Once we are done with estimating the relative position of the images, the 3D position of all corresponding points will be reconstructed by linear triangulation [6], [7], [8]. The final step is doing a refinement process by defining a cost function for any set of parameters (e.g., SEM extrinsic parameters and 3D positions) as to whether this is a good or bad set and find the best fitness model in the set.

Further information about the proposed 3D SEM surface reconstruction framework along with several experimental validations are explained in [2].

**Fig. 2:**
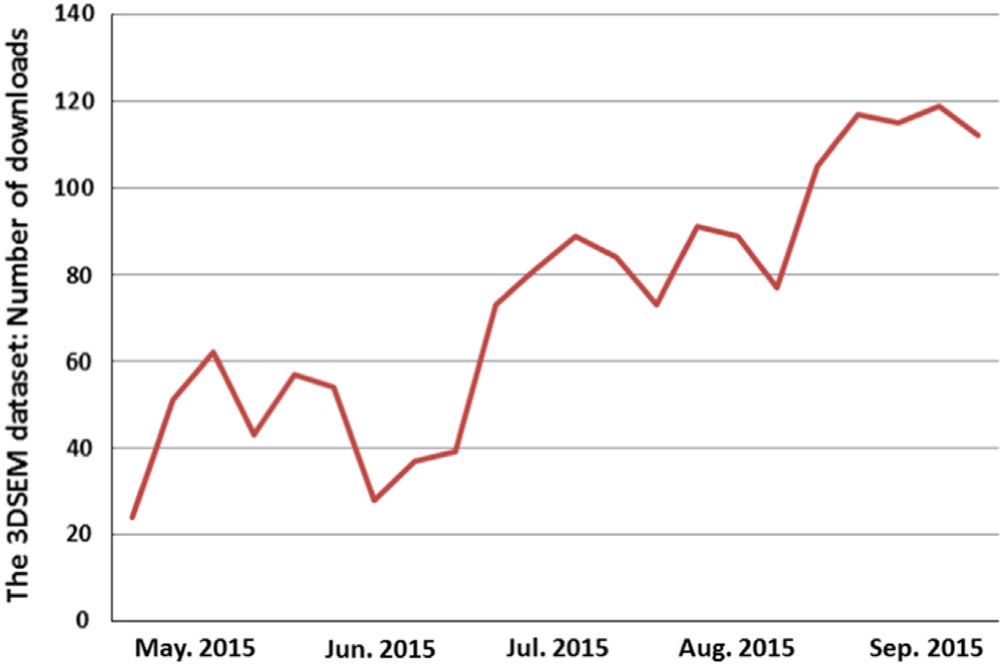
The 3DSEM dataset impact. The figure shows number of times that the dataset has been downloaded since May 2015.

Based on the history of downloads (Fig. 2), the 3DSEM appears to be a popular dataset in the research community. In the near future, we would very much like to increase the number of users for the project and create more datasets using different algorithms.

## Figures and Tables

**Table 1:** Dataset characteristics [3].

## Appendix A Supplementary material

Supplementary material

Supplementary material Several microscopic samples along with their 3D reconstructed surfaces are presented in this video file.

## References

[1] 〈http://www.selibcv.org/3dsem〉.

[2] Tafti Ahmad P., Kirkpatrick Andrew B., Alavi Zahrasadat, Owen Heather A., Yu Zeyun (2015). Recent advances in 3D SEM surface reconstruction. Micron.

[3] 〈http://meshlab.sourceforge.net/〉.

[4] Chandler Douglas E., Roberson Robert W. (2009). Bioimaging: Current Concepts in Light and Electron Microscopy.

[5] Egerton Ray (2006). Physical Principles of Electron Microscopy: An Introduction to TEM, SEM, and AEM.

[6] Hartley Richard, Zisserman Andrew (2003). Multiple View Geometry in Computer Vision.

[7] Cyganek Boguslaw, Siebert J. Paul (2011). An Introduction to 3D Computer Vision Techniques and Algorithms.

[8] Wöhler Christian (2012). 3D Computer Vision: Efficient Methods and Applications.

