# Supplementary figures and images for: 3DSEM: A 3D microscopy dataset
Source: Data Brief. 2015 Dec 2;6:112–6. doi: 10.1016/j.dib.2015.11.018 (PMC4685174; doi:10.1016/j.dib.2015.11.018)

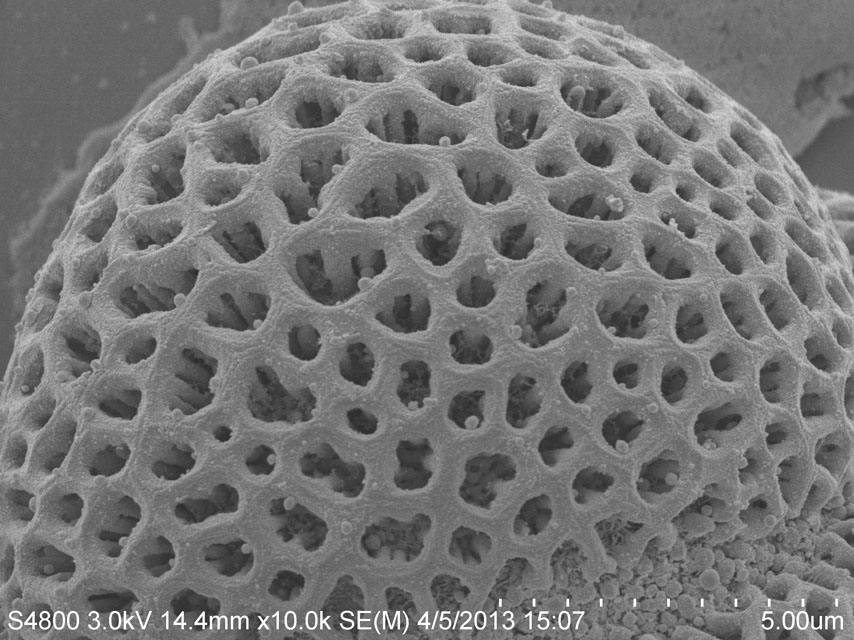

Supplement: Supplementary file 1 — Supplementary material [file mmc1.zip › Dataset/pollen grain from Brassica rapa/2d images/Pollen1001.jpg]

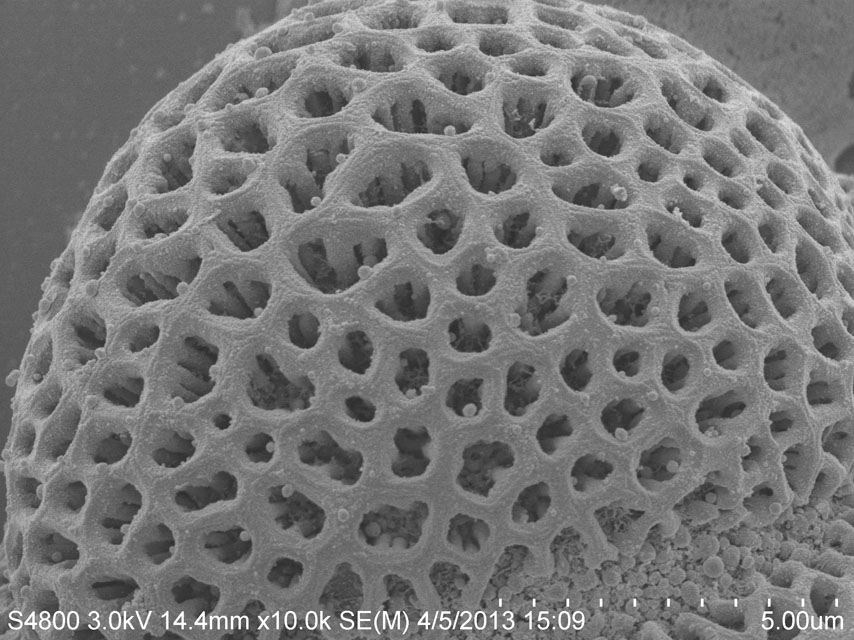

Supplement: Supplementary file 1 — Supplementary material [file mmc1.zip › Dataset/pollen grain from Brassica rapa/2d images/Pollen1002.jpg]

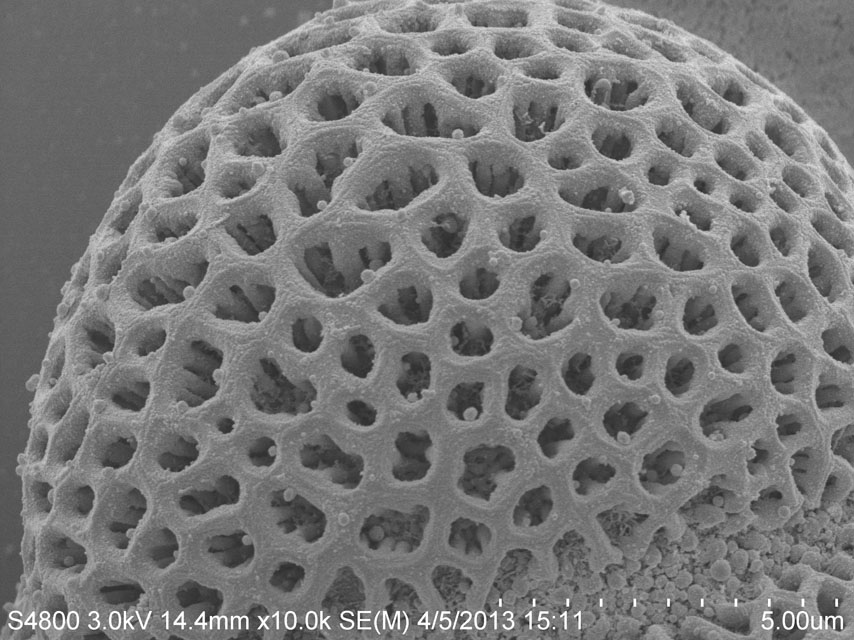

Supplement: Supplementary file 1 — Supplementary material [file mmc1.zip › Dataset/pollen grain from Brassica rapa/2d images/Pollen1003.jpg]

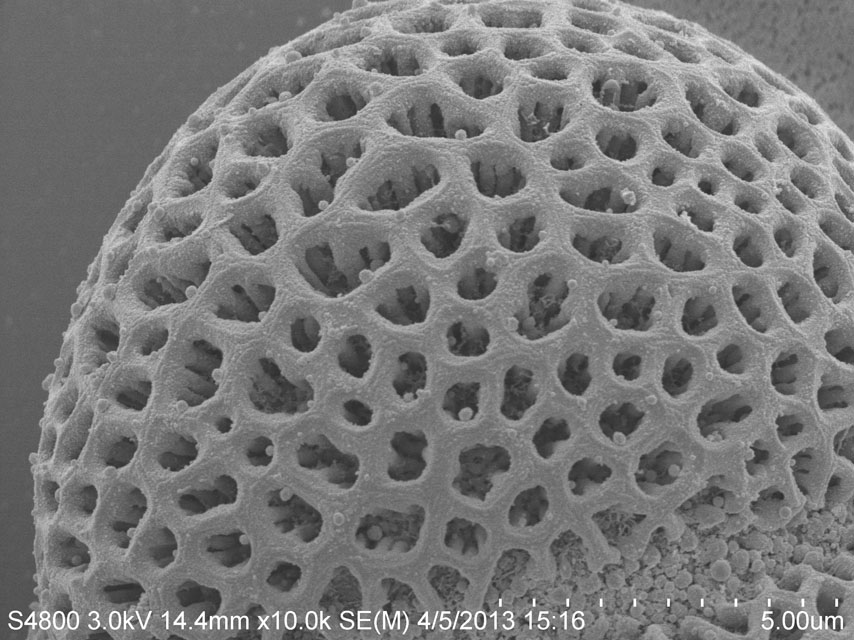

Supplement: Supplementary file 1 — Supplementary material [file mmc1.zip › Dataset/pollen grain from Brassica rapa/2d images/Pollen1004.jpg]

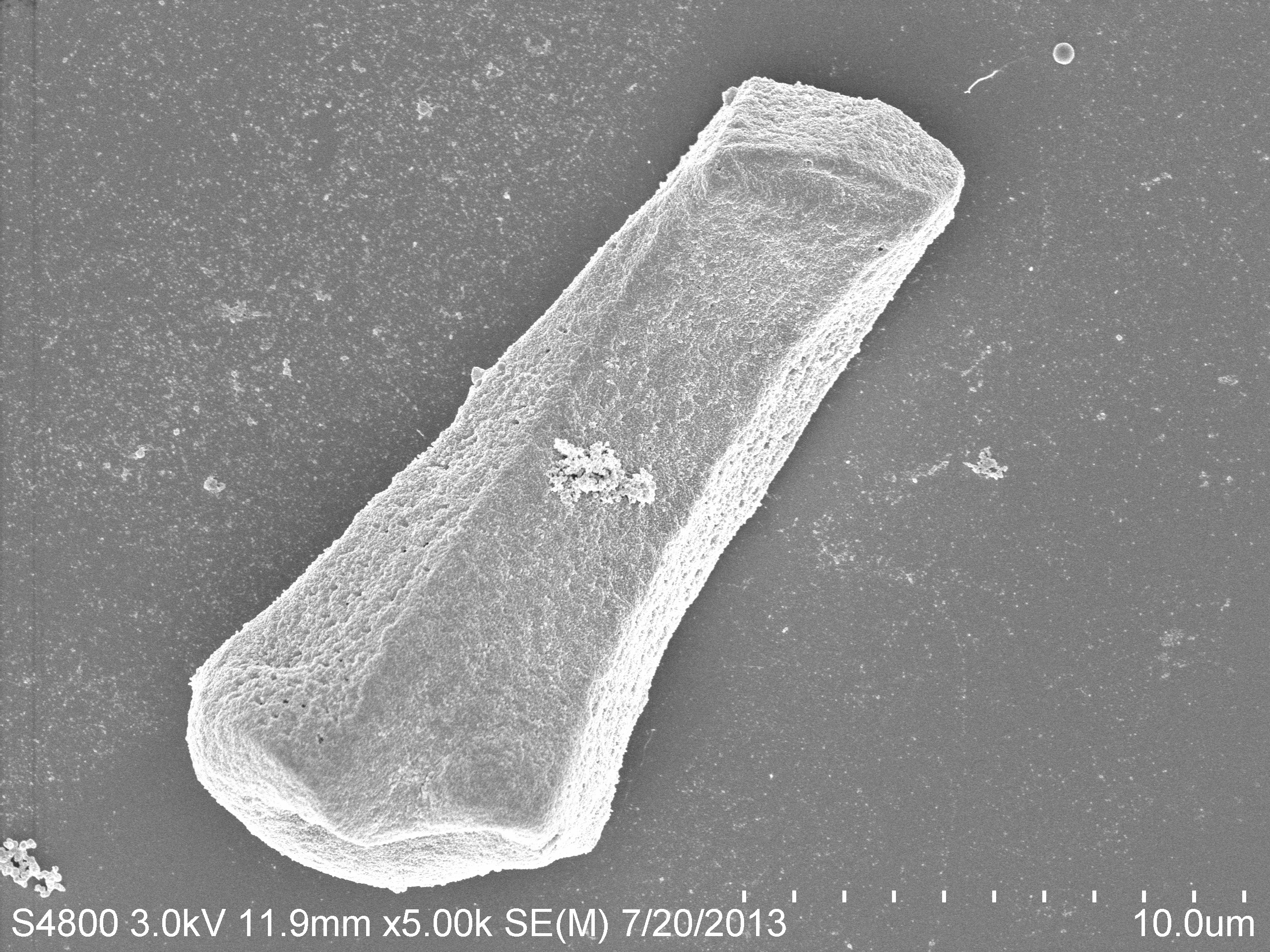

Supplement: Supplementary file 1 — Supplementary material [file mmc1.zip › Dataset/tapetal cell of Arabidopsis thaliana/2d images/Cell2001.jpg]

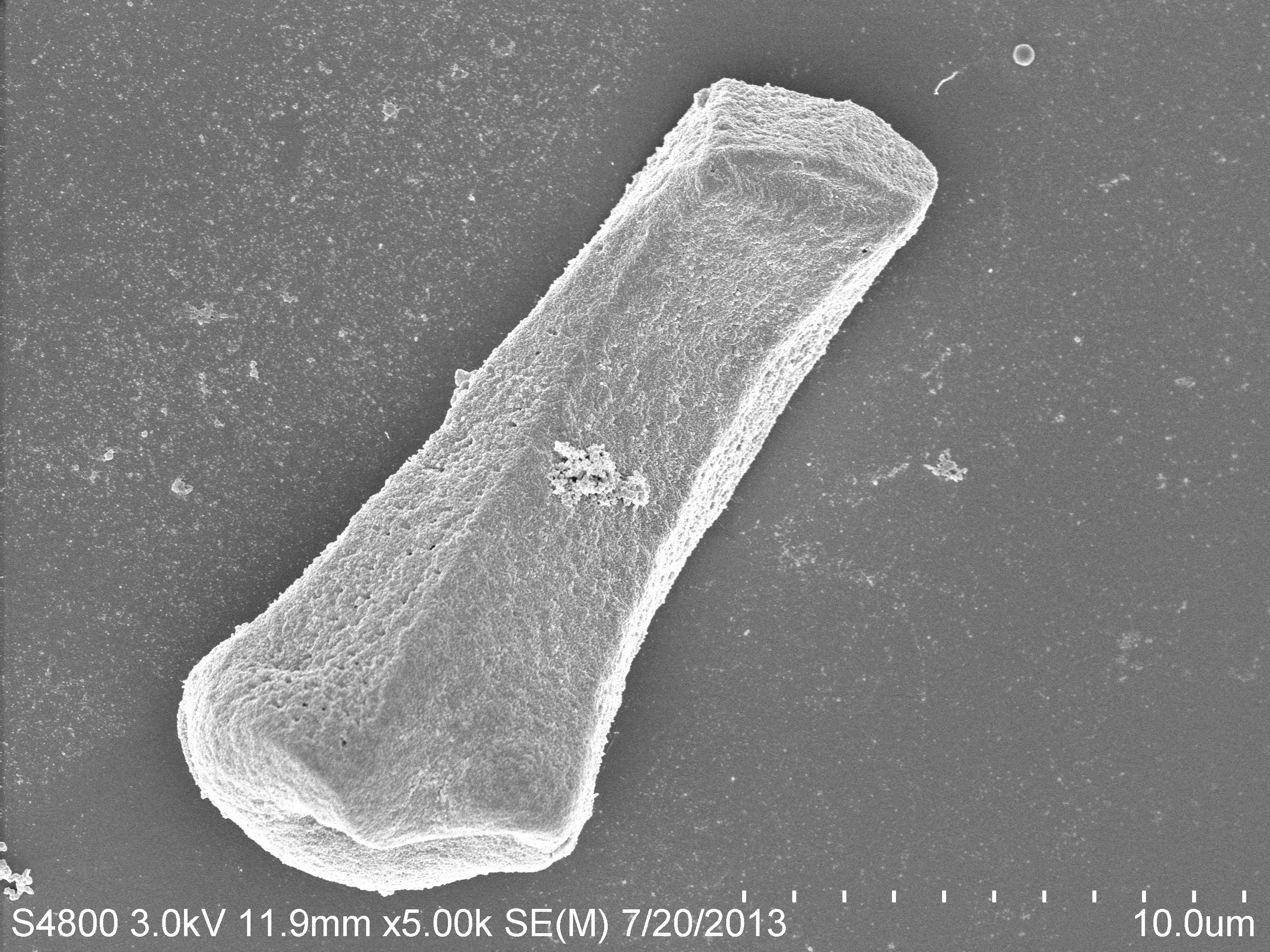

Supplement: Supplementary file 1 — Supplementary material [file mmc1.zip › Dataset/tapetal cell of Arabidopsis thaliana/2d images/Cell2002.jpg]

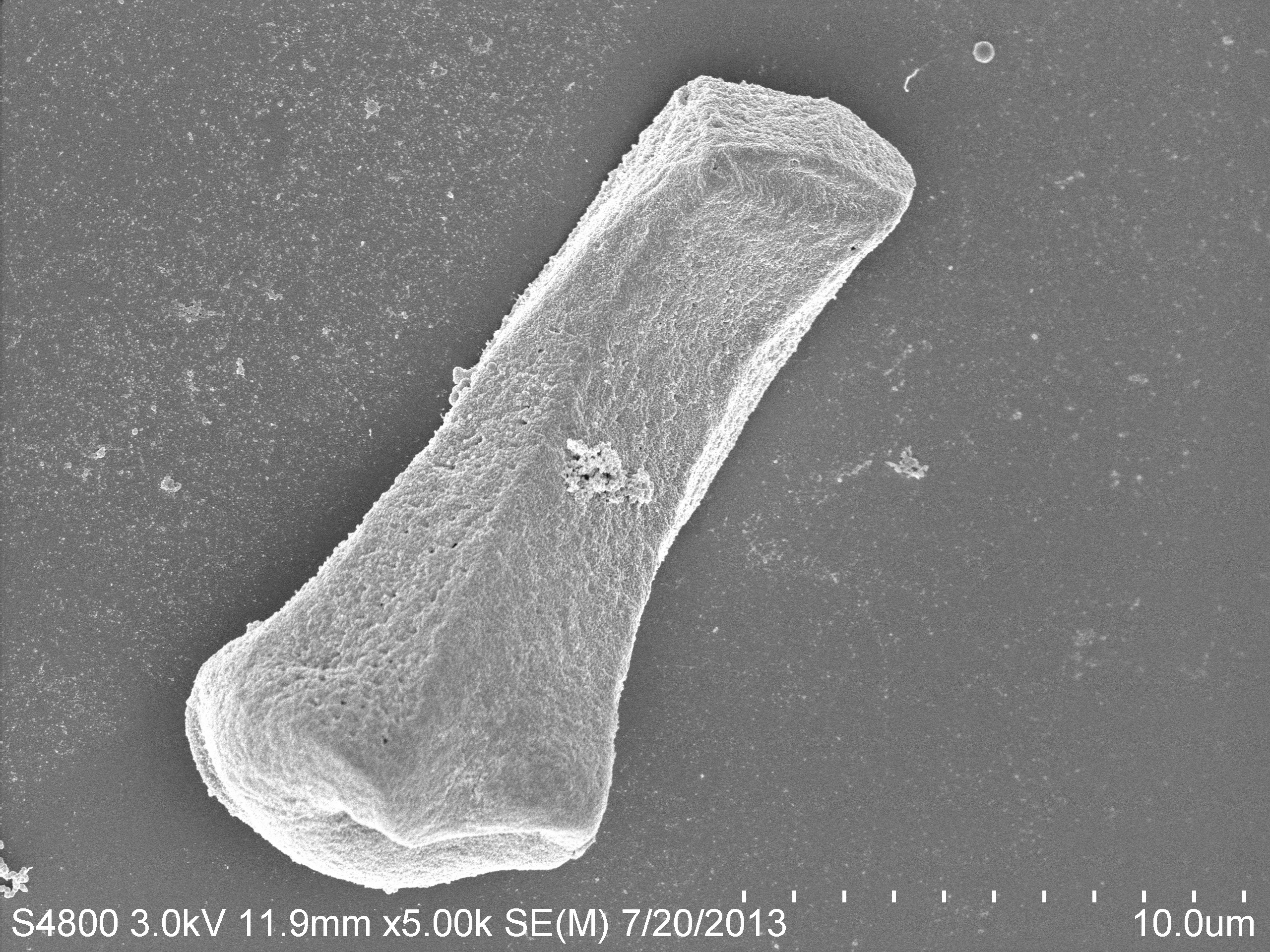

Supplement: Supplementary file 1 — Supplementary material [file mmc1.zip › Dataset/tapetal cell of Arabidopsis thaliana/2d images/Cell2003.jpg]

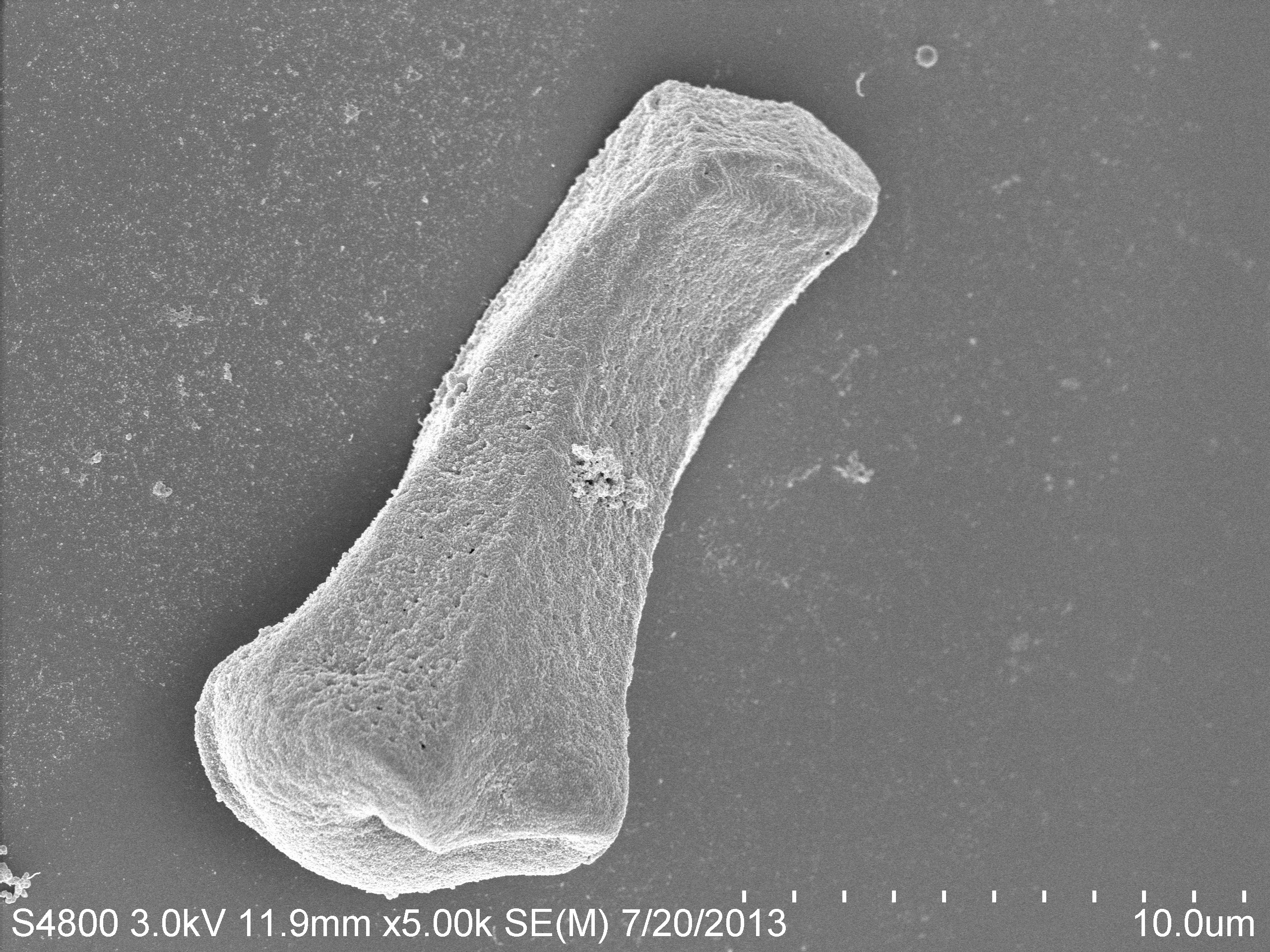

Supplement: Supplementary file 1 — Supplementary material [file mmc1.zip › Dataset/tapetal cell of Arabidopsis thaliana/2d images/Cell2004.jpg]

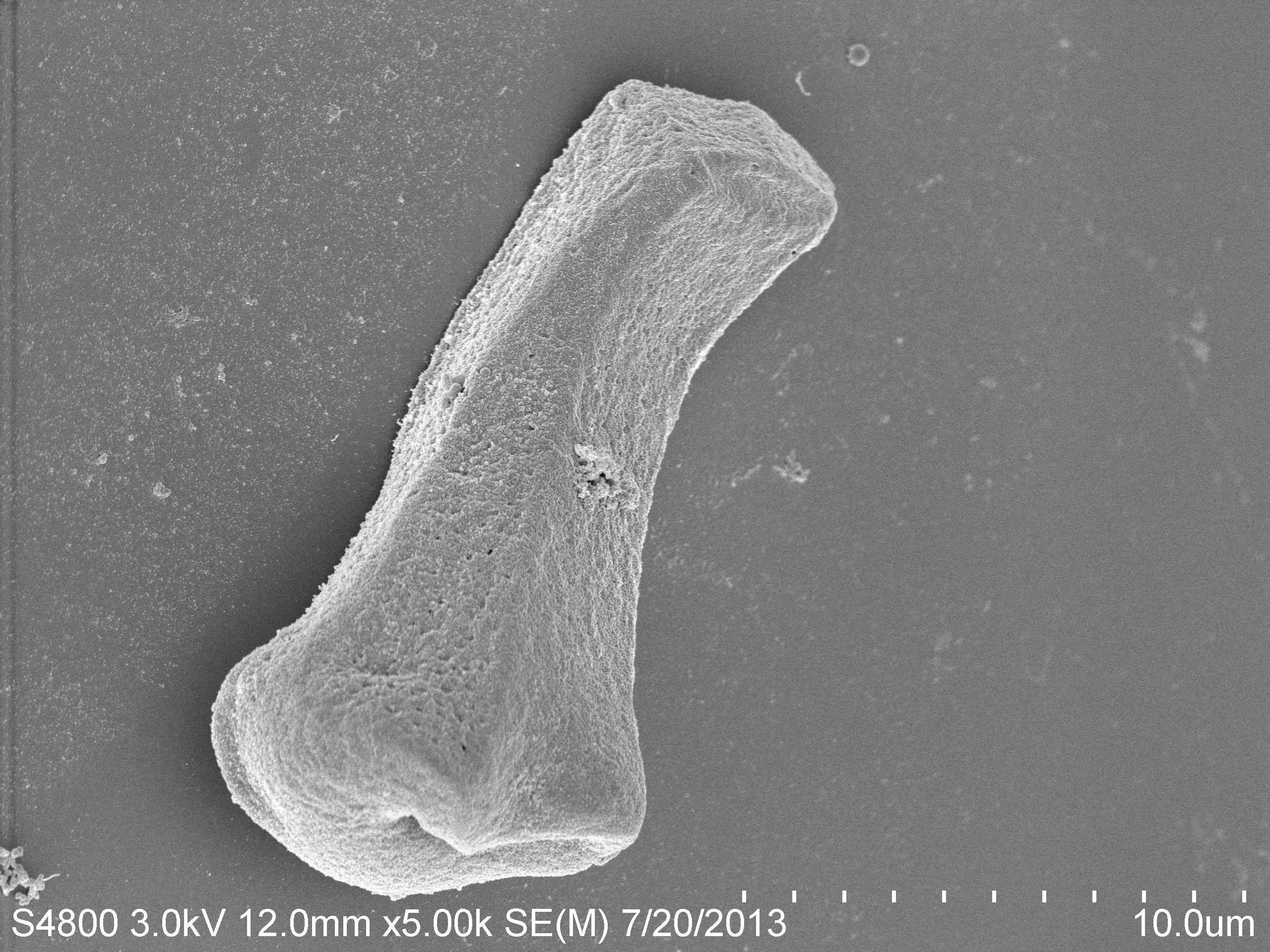

Supplement: Supplementary file 1 — Supplementary material [file mmc1.zip › Dataset/tapetal cell of Arabidopsis thaliana/2d images/Cell2005.jpg]
